# On decreasing inequality in health care in a cost-effective way

**DOI:** 10.1186/1472-6963-14-79

**Published:** 2014-02-20

**Authors:** Antti Malmivaara

**Affiliations:** 1 Centre for Health and Social Economics, National Institute for Health and Welfare, Mannerheimintie 166, 00270 Helsinki, Finland

**Keywords:** Benchmarking, Cost-effectiveness, Evidence based medicine, Inequality, Medical ethics, Quality improvement, Real effectiveness medicine, Socioeconomic status

## Abstract

**Background:**

Scientific evidence indicates that access to health care services shown effective is often poorer among the disadvantaged people than among the non-disadvantaged. Advancing cost-effectiveness and equality in obtaining services shown effective are often stated as the two main objectives for health care.

**Discussion:**

The important question is how to decrease inequality in a most cost-effective way. Some evidence indicates that effectiveness and cost-effectiveness of interventions may be high among the disadvantaged patient groups. However, the relationship between decreasing inequality and cost-effectiveness is complex and depend e.g. on clinical and health care system factors.

**Summary:**

To expand the knowledge base for advancing equality in a cost-effective way, scientific efforts on four levels are suggested: research into ways how practitioners can provide efficient services for the disadvantaged patients, widening of scope at all levels of evidence based medicine to account also for the disadvantaged, research on quality improvement and evidence implementation, as well as benchmarking research including documentation of services among the disadvantaged.

## Background

Inequality in health care is defined in this article as inferior access to or inferior quality (diagnostic and treatment procedures) of *services shown effective* (in randomised controlled trials or based on wide consensus among experts – e.g. total hip or knee replacement) among people belonging to some disadvantaged patient group. The disadvantage may be due to age, culture, education, ethnic origin, gender, occupation, place of residence, race, religion, social capital or socioeconomic status [1].

There is evidence that disadvantaged people have on average poorer access to services, and once they have an access to service they get less often treatments that have been shown effective in randomized trials, and in case they obtain these treatments the quality they receive is poorer than among people not belonging to these groups [2-5]. Partly as a consequence of poorer access and non-optimal quality of the services, prognosis of these patients may be inferior to that of the well to do patients [6].

There is some evidence from randomized trials and systematic reviews that effectiveness of an intervention may be even high among the most disadvantaged, and that the cost-effectiveness of the intervention may be good [7,8]. In some cases cost-effectiveness of an intervention is better when resources are allocated to the poorest and most marginalised populations than when allocating to those doing better [9]. However, in some cases the additional cost of tailor-made interventions for the disadvantaged may be high in comparison to benefits [10]. Unfortunately, direct evidence comparing effectiveness and cost-effectiveness of a specified intervention for a particular disorder among the disadvantaged and non-disadvantaged populations is quite limited.

The horizontal equity principle of providing equal treatment for equal medical need [11] is employed in this article, but within this framework the focus is in those interventions considered effective based on scientific evidence or wide consensus among medical experts. The access in this article refers particularly to opportunities and not to utilization itself, as the latter can differ between population groups also due to reasons beyond the influence of the health care system [12]. The question of distribution of health across different groups of society is not considered, because the potential of health care system is limited in this respect [13]. The conceptual categories of questions regarding measurement techniques including inequity indices are not covered.

The question of whether decreasing of inequality would lower, maintain or increase overall cost-effectiveness of the health care system is not considered, and neither the efficiency/equity trade-off [14]. An unbiased assessment of each particular patient-intervention context would require a systematic review of its own.

The aim of this paper is to consider ways how to promote scientific evidence on decreasing inequality in a cost-effective manner, and how to use this evidence in advancing equality and cost-effectiveness in health care.

## Discussion

### How to increase scientific evidence and promote equality and cost-effectiveness

Evidence of advancing equality in a cost-effective manner could be promoted by efforts at four levels: clinical expertise, scientific evidence, quality improvement and benchmarking [15].

Firstly, there is a need for both quantitative and qualitative research on how to teach and promote know-how of health care personnel for cost-effective treatment among the disadvantaged. The know-how includes understanding of etiology, pathogenesis, diagnosis and treatment of diseases combined with practical experience and expertise on how to deal with the disease among the disadvantaged. Special expertise may be needed as e.g. cultural differences may have impact on adherence to treatment regimens. Moreover, acceptability of effective services provided by the health care system may differ across population groups, and should also be considered. In a systematic review strategies to increase treatment adherence show moderate effectiveness and cost-effectiveness [16]. The CanMeds framework aiming to advance the abilities of health care professionals to reach optimal patient outcomes can be utilized and further tested in scientific studies [17].

Secondly, scientific goals at all levels of evidence based medicine (randomized controlled trials, systematic reviews, health technology assessments, clinical guidelines) are suggested to include cost-effectiveness of interventions also among the disadvantaged. There are large deficiencies in the scientific evidence of what is the effectiveness and cost-effectiveness of interventions among the disadvantaged groups.

The exclusion criteria in randomized trials are often such that some of the disadvantaged groups will not be included and thus no effectiveness data will emerge [18]. The CONSORT guideline for reporting randomized controlled trials does not include an item on disadvantaged patient groups [19]. This poses a problem in creating new evidence applicable to the disadvantaged patient groups.

Systematic reviews have rarely considered the disadvantaged groups: only 1% of a random sample of Cochrane reviews assessed differences in effectiveness of interventions across socioeconomic or demographic factors [20]. Thus, systematic reviews currently provide very little data applicable to the disadvantaged patient groups [21]. Applaudingly, a recent recommendation on how to include these patient groups in systematic reviews has been launched [22]. Hopefully this recommendation will be widely adopted by organizations and individuals undertaking systematic reviews.

The clinical guidelines do not usually consider the disadvantaged patient groups when assessing effectiveness or safety of interventions [23]. The AGREE instrument guiding appropriate conduct of clinical guidelines does not include the disadvantaged groups [24]. An authoritative international recommendation of how to include the disadvantaged patient groups in clinical guidelines is suggested.

Thirdly, it is suggested that quality improvement and implementation science would always consider also questions concerning services for the disadvantaged. There is a need for research assessing whether treatments suggested by evidence-based guidelines are actually implemented in ordinary clinical practice throughout the population. For example, in a study utilizing the population-based South London Stroke Register, the appropriateness of treatment had improved considerably from 1995 to 2009, but the implementation of evidence-based care was still not optimal, and there were inequalities between socioeconomic groups [2]. The optimal strategies targeted to the developed and developing countries should also be explored.

Fourthly, benchmarking of services between providers and geographical areas are suggested [25]. Optimally the benchmarking would be accompanied by scientific research on access, quality, costs and effectiveness of services for all patient groups including the disadvantaged. As the number of high quality benchmarking studies is so far small, promotion of this type of research is suggested.

## Summary

High quality services are provided by competent professionals who make use of the best current scientific evidence with an aim to maximize effectiveness and minimize harm. The lost opportunities for providing effective services for the disadvantaged patient groups may lessen cost-effectiveness of the health care systems as a whole, but also of any particular health care provider who fails to provide effective services for the whole population which it serves. Poor safety of the services provided for the disadvantaged patient groups will further decrease the cost-effectiveness of interventions. In order to decrease the equity gap when extending the scientific evidence of efficacy into effectiveness in ordinary health care settings, the equity effectiveness loop can be utilized [26].

There is a vast evidence showing widespread inequality in obtaining services shown scientifically effective, particularly in the developing countries but also in the developed ones [3]. The current relevance of advancing equality is emphasized by evidence from developed countries that socioeconomic inequality may be widening further, shown e.g. in mortality amenable to health care interventions [27].

There are certainly differences in the relationship between decreasing inequality and increasing cost-effectiveness. The association will depend on properties of the health care systems, the wealth of the society, on the degree of inequality in a particular country, and on the clinical context. The latter can be made explicit with the PICO (patient, intervention, comparator, outcome) -concept: in some diseases the cost-effectiveness of a particular intervention for the disadvantaged patients will be better, while in other cases similar or even worse than among the well to do people. In the latter cases better strategies for providing effective treatment for the disadvantaged patients may increase effectiveness [16]. In an experimental study low-income persons who had won in a lottery the Medicaid insurance for obtaining health care services were compared with those who failed to win: the persons who did win the insurance had better access and utilization of health care services and substantial improvements in mental health, but despite better diagnosis of diabetes no reduction in glycated haemoglobin levels [28]. These findings point out that access to services as such may provide only limited benefits for the disadvantaged persons.

To sum up, this paper suggests a framework for research and practice on how to allocate the limited resources in most cost-effective way encompassing also those who currently do not obtain the services shown effective. The method is suggested to be used for advancing both the ethical objectives and cost-effectiveness requirements of the health care system. Research and development activities at the four scientific and practical levels are suggested.

## Competing interests

I declare that I have no conflict of interests.

## Pre-publication history

The pre-publication history for this paper can be accessed here:

http://www.biomedcentral.com/1472-6963/14/79/prepub
